# Tumor Treating Fields for Glioblastoma Therapy During the COVID-19 Pandemic

**DOI:** 10.3389/fonc.2021.679702

**Published:** 2021-05-07

**Authors:** Na Tosha N. Gatson, Jill Barnholtz-Sloan, Jan Drappatz, Roger Henriksson, Andreas F. Hottinger, Piet Hinoul, Carol Kruchko, Vinay K. Puduvalli, David D. Tran, Eric T. Wong, Martin Glas

**Affiliations:** ^1^ Division of Neuro-Oncology, Department of Neurology, Geisinger Health, Neuroscience & Cancer Institutes, Danville, PA & Geisinger Commonwealth School of Medicine, Scranton, PA, United States; ^2^ Neuro-Oncology, Banner MD Anderson Cancer Center, Phoenix, AZ, United States; ^3^ Department of Population and Quantitative Health Sciences, Case Western Reserve University School of Medicine & Research and Education, University Hospitals of Cleveland, Cleveland, OH, United States; ^4^ Hillman Cancer Center, Department of Medicine and Neurology, University of Pittsburgh, Pittsburgh, PA, United States; ^5^ Department of Radiation Sciences & Oncology at the University of Ume, Ume, Sweden; ^6^ Departments of Clinical Neurosciences & Oncology, Lausanne University Hospital (CHUV), Lausanne, Switzerland; ^7^ Global Medical Affairs, Novocure Inc., New York, NY, United States; ^8^ Central Brain Tumor Registry of the United States (CBTRUS), Hinsdale, IL, United States; ^9^ Department of Neuro-Oncology, The University of Texas MD Anderson Cancer Center, Houston, TX, United States; ^10^ Lillian S. Wells Department of Neurosurgery and Preston A. Wells, Jr. Brain Tumor Center at the McKnight Brain Institute of the University of Florida College of Medicine, Gainesville, FL, United States; ^11^ Department of Neurology, Beth Israel Deaconess Medical Center, Harvard Medical School, Boston, MA, United States; ^12^ Division of Clinical Neurooncology, Department of Neurology and German Cancer Consortium (DKTK) Partner Site, University Hospital Essen, University Duisburg-Essen, Essen, Germany

**Keywords:** COVID-19, tumor treating fields, glioblastoma, recurrent glioblastoma, elderly

## Abstract

**Background:**

The COVID-19 pandemic has placed excessive strain on health care systems and is especially evident in treatment decision-making for cancer patients. Glioblastoma (GBM) patients are among the most vulnerable due to increased incidence in the elderly and the short survival time. A virtual meeting was convened on May 9, 2020 with a panel of neuro-oncology experts with experience using Tumor Treating Fields (TTFields). The objective was to assess the risk-to-benefit ratio and provide guidance for using TTFields in GBM during the COVID-19 pandemic.

**Panel Discussion:**

Topics discussed included support and delivery of TTFields during the COVID-19 pandemic, concomitant use of TTFields with chemotherapy, and any potential impact of TTFields on the immune system in an intrinsically immunosuppressed GBM population. Special consideration was given to TTFields' use in elderly patients and in combination with radiotherapy regimens. Finally, the panel discussed the need to better capture data on COVID-19positive brain tumor patients to analyze longitudinal outcomes and changes in treatment decision-making during the pandemic.

**Expert Opinion:**

TTFields is a portable home-use device which can be managed via telemedicine and safely used in GBM patients during the COVID-19 pandemic. TTFields has no known immunosuppressive effects which is important during a crisis where other treatment methods might be limited, especially for elderly patients with multiple co-morbidities. It is too early to estimate the full impact of COVID-19 on the global healthcare system and on patient outcomes and the panel strongly recommended collaboration with existing cancer COVID-19 registries to follow CNS tumor patients.

## Introduction

The global case-fatality ratio for COVID-19 in confirmed cases was 2.1% as of January 11, 2021 (1), and the rate increases to 22.4% in the cancer population (2). GBM patients are considered a vulnerable patient population during the ongoing COVID-19 pandemic mainly due to the increased incidence of GBM in the elderly population (3), treatment related immunosuppression, and the requirement for frequent hospital visits. Importantly, the >65-year-old age group is expected to increase over the next two decades in the USA, Canada, Australia, and Europe (4). Various groups of experts have already published recommendations and considerations concerning the treatment of patients with high grade glioma during the early stages of the COVID-19 pandemic (5, 6). Elderly patients have a significantly higher risk of mortality when infected with COVID-19 (7) as do patients with multiple co-morbidities, common in the elderly population (8), especially obesity and hypertension (9,11).

Recently published recommendations for care of brain tumor patients with COVID-19 focus on the need to continue essential treatments such as surgery, but to carefully assess the need for full cycles of radiation therapy as well as the timing of immunosuppressive agents such as temozolomide (TMZ) and steroids (5, 6). Strict adherence to physical distancing rules is reinforced by these recommendations as the safety of both patients and health care providers is of utmost priority. As such, in-person patient visits to health care facilities should be reduced to a safe minimum to minimize potential exposure of the patient and to ensure adequate safety of the ongoing treatment.

The aforementioned recommendations to treat patients with brain tumors in the context of the COVID-19 pandemic (5, 6) focused on general recommendations and not on specific therapies such as TTFields. TTFields is an established treatment modality for newly diagnosed GBM, the most common type of primary malignant brain tumor in adults (3), and is delivered using Optune, a portable home-use medical device. TTFields are low intensity, intermediate frequency (200 kHz) alternating electric fields that disrupt cancer cell division (12, 13). The large Phase 3 randomized control trial, EF-14, has demonstrated TTFields efficacy for GBM: TTFields combined with TMZ significantly increased overall survival *vs* TMZ alone in patients with newly diagnosed GBM (14) without deterioration in quality of life (QoL) (15).

**Figure 1:**
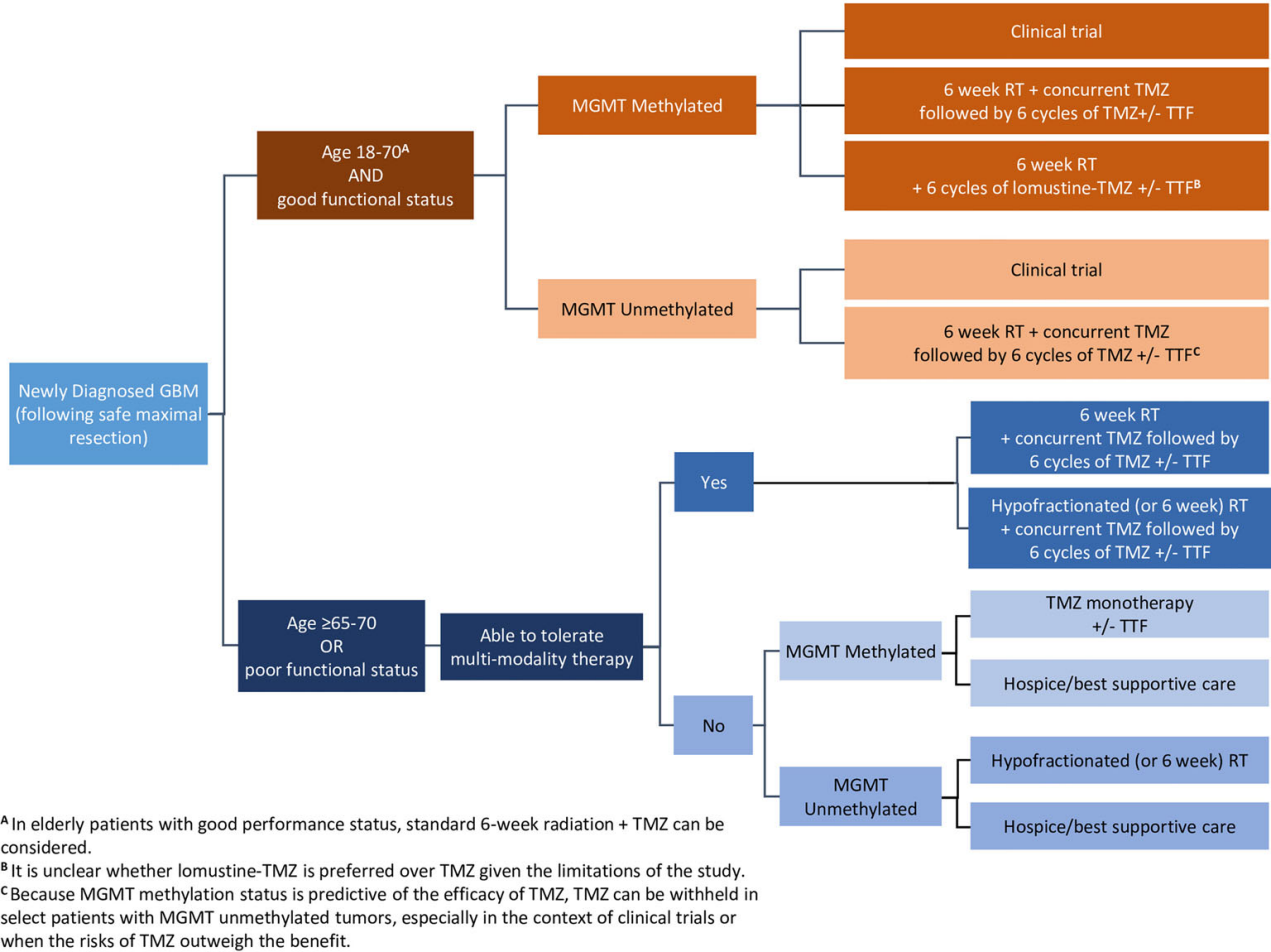
Wen, etal. Adult glioblastoma management: a Society for Neuro-Oncology (SNO) and European Society of Neuro-Oncology (EANO) consensus review. Neuro-Oncology. 2020;22(8):10731113. DOI: 10.1093/neuonc/noaa106*. Adapted and* r*eprinted by permission of Oxford University Press on behalf of the Society for Neuro-Oncology.* GBM, glioblastoma; MGMT, O(6)-methylguanine-DNA methyltransferase; RT, radiation therapy; TMZ, temozolomide; TTF, Tumor Treating Fields. *Disclaimer: OUP andSNOare not responsible or in any way liable for the accuracy of the adaptation.Licenseeis solely responsible for the adaptation in this publication/reprint.*

The recently published SNO/EANO consensus article (16) summarizes the role of TTFields in newly diagnosed GBM patients aged 18 to 70 years with good functional status as compared to poor performing newly diagnosed GBM patients aged 65 to 70 years, and evaluated both MGMT-methylated and unmethylated patients in both groups (**Figure 1**).

TTFields has no known suppressive effect on the immune system, and may be a reliable treatment modality in times of a health pandemic when other treatment methods that require in-person visits to the hospital/doctor are limited. In-person monthly visits by device support specialists (DSS) are critical to provide patient-education and training for proper use of new and replacement equipment, array placement, and appropriate follow-up on usage. These practices required review during the pandemic to better comply with physical distancing recommendations. Bernhardt et al. noted that TTFields therapy regimen is generally associated with a low relative likelihood of having viral exposure. (5) Still, device delivery and support has been transitioned to include virtual and telemedicine practices which allow for safer physical distancing with fewer in-person encounters.

International experts in the field of neuro-oncology with TTFields experience were convened by Novocure to provide guidance and discuss available data on TTFields use in both newly diagnosed GBM and recurrent GBM in the context of the COVID-19 pandemic. This paper provides an opinion from an expert panel for a risk-benefit based decision regarding the inclusion of TTFields therapy to treat GBM during the COVID-19 pandemic.

## Methods

### Expert Panel Discussion

A virtual meeting with a panel of multinational experts was conducted on May 9, 2020. The panel was chosen by specialists in GBM treatment with TTFields experience, and consisted of 7 neuro-oncologists (including two internal medicine specialists and one surgeon), an epidemiologist, a radiation-oncologist, President of the Central Brain Tumor Registry of the United States (CBTRUS), and Novocures Head of Global Medical Affairs. In addition to the panel members, further discussions were held with other researchers, statisticians, and COVID-19 cancer registry developers relevant to the neuro-oncology community after the advisory board meeting.

### Objectives of the Panel Discussion

The purpose of this expert panel discussion was to gain insights on:

1. Treatment challenges and selection during the COVID-19 pandemic
2. Safety profile of TTFields as observed by the attendees
3. Clinical and patient experiences using TTFields during the pandemic
4. Opportunities to expand awareness and education on TTFields safety profile
5. TTFields use in clinical trials during the pandemic
6. Future directions in research and treatment guidelines in cancer COVID-19 patients

## Discussion

### TTFields Impact on the Immune System

Given that GBM patients are in a baseline immune compromised state (17) and undergo additional immune suppression related to standard of care chemo- and radiotherapies, it was noted that the use of adjuvant TTFields therapy poses no known additional risk. Recent preclinical investigations have demonstrated that TTFields induced immunogenic (tumor) cell-death and potentially enhances the anti-tumor effects of the anti-PD-1 immune checkpoint inhibitor when used in combination (18). There were no observed negative in vivo effects on immune system function in tested mouse models (18). In these experiments, pulmonary regions were treated with TTFields and the frequency of T-cells (CD8+, Tregs, CD4+), dendritic cells, and macrophages were investigated (18). Hematopoiesis has been demonstrated to be unaffected by TTFields as hematopoietic stems cells, residing in the bone marrow, are shielded due to the high bone impedance resulting in significantly lower field intensities (19). Diamant *et al.* reported no impact on T-cell counts secondary to TTFields (20). In vitro studies of T-cell functionality such as induced cytotoxic degranulation and direct cytotoxic activity were not inhibited by TTFields (20). Taken together, the preclinical data, to date, suggests that TTFields do not induce local and systemic immunosuppressive effects and supports a favorable clinical safety profile for TTFields. The combination of TTFields with an immune-checkpoint inhibitor (anti PD-1) and TMZ is currently under investigation for newly diagnosed GBM (2-THE-TOP trial) (21), and available preliminary safety results suggest that the combination therapy is well tolerated.

### Concomitant Use of Chemotherapy and TTFields

GBM patients may be intrinsically immunosuppressed as demonstrated by reduced CD4/CD8 counts (22). Standard treatments such as TMZ, dexamethasone, and lomustine (CCNU, Gleostine) have been reported to further hamper the adaptive immune system by reducing the CD4/CD8 counts (23, 24). Importantly, the CeTeG trial evaluated combined use of TMZ and CCNU without noting increased infection rates (25), which challenges the overall immunosuppressive impact of these chemotherapy regimens. However, immune competence against infection may have more to do with the innate than the adaptive immune system (26, 27). While the benefit of TMZ in MGMT-promoter-unmethylated patients has been debated (28), many have continued its use in this patient population despite pandemic concerns. Overall, the pandemic has not driven indications for major modifications in the use of standard chemotherapeutic regimens in GBM care.

The randomized phase III EF-11 trial investigated safety of TTFields as monotherapy (n=116) in recurrent GBM patients compared to best standard of care (BSC; n=91). Patients receiving TTFields in the EF-11 trial had fewer blood and lymphatic system disorders (4.3% TTFields alone *versus* 18.7% BSC), infections (4.3% TTFields alone *versus* 12.1% BSC) and respiratory, thoracic, and mediastinal disorders (6.0% TTFields alone *versus* 11.0% BSC) (29, 30). Of note, the EF-14 trial demonstrated similar toxicity profiles between the TTFields plus second-line therapy *versus* second-line therapy alone following the first progression (31). In addition to the phase III clinical trials, retrospective investigations of TTFields in combination with other therapies (i.e. TMZ and CCNU) found no increase in adverse events (AE) in newly diagnosed GBM (32) nor in TTFields plus CCNU in recurrent GBM (33).

In recurrent GBM (TTFields used as monotherapy) (30) and newly diagnosed GBM (TTFields plus TMZ) (14), the most common TTFields-related AE was skin reaction beneath the arrays, with no significant increase in systemic AEs, including blood and lymphatic system disorders. In many cases, skin reaction can be prevented and managed at home via telemedicine assessment and appropriate use of topical therapies as noted in the skin reaction guidelines (34). Therefore, treatment of skin reactions can take place in compliance with physical distancing regulations. Additionally, patients who receive TTFields treatment consistently report no deterioration in quality of life (15, 30).

### TTFields in Elderly Patients

In a recent real world evidence study in England covering approximately 40% of the population, increasing age was strongly associated with risk of COVID-19related mortality (fully adjusted hazard ratios per age group: 40 to 49 years, 0.3 *vs* 50 to 59 years, 1.0 *vs* 60 to 69 years, 2.4 *vs* 70 to 79 years, 6.1 vs 80+ years, 20.6) (7). The efficacy of TTFields in elderly newly diagnosed GBM patients (age 65) was also investigated in the EF-14 trial, and this subgroup analysis indicated that TTFields in combination with TMZ (n=89) was associated with significantly increased survival compared to TMZ alone (n=45) (17.4 months versus 13.7 months; HR, 0.51; 95% CI, 0.330.77) (14). Both data from the EF14 study (n=134) (35) as well as the recently published global post-marketing surveillance analysis of TTFields (n=2,887 elderly patients out of 11,029 total patients) (36) demonstrated a comparable safety profile between the elderly subgroup and non-elderly subgroups treated with TTFields plus TMZ. Specifically, the most common TTFields-related AE, skin reaction, was comparable in elderly, adult, and pediatric subgroups, with an incidence of 36%, 34%, and 37% respectively. The incidence of infections was <1% in all groups (36), and so addition of TTFields in high-risk elderly GBM patients is not expected to be associated with poorer outcomes in the context of COVID-19.

### TTFields and Radiation Therapy

Use of TTFields with radiation induced cellular DNA damage demonstrated synergistic effects *in vitro* (37, 38). Currently, TTFields is approved for use in newly diagnosed GBM in combination with adjuvant TMZ initiated after completion of chemoradiation (i.e. in concert with the current GBM standard of care). There was agreement among the experts that there is no objective data to suggest that radiation dose or schedule negatively impacts the efficacy of TTFields and, presumably, TTFields could also be offered after hypofractionated chemoradiotherapy when indicated.

A pilot trial by Bokstein et al. (39) demonstrated the feasibility and safety of TTFields administered concurrently with radiotherapy and TMZ in newly diagnosed GBM patients. Several ongoing newly diagnosed GBM clinical trials are investigating the safety and efficacy of this concurrent triple-modality therapy, and include: (1) the phase II study conducted in Israel (40), (2) the German PriCoTTF phase II study (41), and (3) the global phase III study EF-32/TRIDENT (42). A phase II study, GERAS, will enroll elderly patients, who will receive TTFields and concomitant hypofractionated radiation therapy and will provide additional insights into the use of TTFields in this patient population (43).

### TTFields Therapy Delivery and Support During the COVID-19 Pandemic

Patients on TTFields undergo routine home-based technical support and education at the start of TTFields and follow up with monthly home-based visits for usage downloads. A recent publication raised important concerns regarding the potential risk of DSS breach of pandemic physical distancing restrictions during these patient interactions (5). This panel reaffirmed the importance of minimizing exposure risks to COVID-19 for both patients and support specialists and supports the measures to reduce infectious exposure risks, such as: (1) DSS to offer virtual treatment starts; (2) DSS to follow strict protocols for wearing full personal protective equipment (PPE) including mask, glasses, gloves, and a disposable gown for all necessary in-person visits; (3) Allowing temporary approval to obtain verbal patient consent via telemedicine for TTFields therapy; (4) Adapting the monthly DSS visit to be completed virtually with device replacements either by shipping (in the US) or non-contact home front door delivery (in Europe); and (5) Availability of new software in the US, called MyLink, which enables patients to remotely download monthly usage reports.

### TTFields and Conduct of Clinical Trials During the COVID-19 Pandemic

The experts indicated that some medical centers have seen an increase in TTFields acceptance and use due to limitations for patient enrollment in many non-TTFields clinical trials. The expert panel agreed that TTFields provides safe and effective practices for newly diagnosed as well as established and recurrent GBM patients on clinical trials which limits exposure risks during the COVID-19 pandemic. The fact that TTFields treatment can be initiated and maintained at home was identified as an advantage when comparing TTFields-based GBM clinical trials with other treatment modalities which might require the in-person patient visits to medical facilities. An expert panelist involved in ongoing TTFields-based clinical trials noted its recommendation for continued recruitment based on the overall favorable safety profile of TTFields.

### Cancer COVID-19 Pandemic Registries and Brain Tumor Patients

Multiple national and international COVID-19 registries have been established since the onset of the COVID-19 pandemic. Important to this effort are the cancer and COVID-19 registries aimed at assessing epidemiological, demographic, and practice and treatment outcomes as well as identifying cancer health disparities uncovered by the COVID-19 pandemic; CCC19 (44), NCI NCCAPS (45), ASCO (46), and ESMO-CoCARE (47). There is some degree of overlap within these registries, but each has specific regulatory constraints and data collection objectives which increases the potential for wide variability in data reporting.

Our expert panel discussed the potential for better alignment with these registries, aiming to increase the representation of central nervous system tumor patients and garner participation from the pertinent neuro-oncology community. Several experts have worked closely with the COVID-19 and Cancer Consortium (CCC19) (44) to begin evaluating the COVID-19 impact on brain tumor patient care and outcomes. The CCC19 (North America) is one of the largest inclusive cancer COVID-19 registries and has partnered with the European Society for Medical Oncology Registries (ESMO-CoCARE (47), covering Europe and Asia) to increase the rate of data collection by combining coverage of their respective participating sites across the world.

## Conclusion

TTFields is a treatment modality for newly diagnosed and recurrent GBM that can be safely administered during the current COVID-19 pandemic when other treatment methods are limited. Since TTFields has no known suppressive effect on the immune system and is an established treatment modality with a proven safety profile, it should be considered in times when immunosuppression and other point of care critical factors such as physical distancing and travel reduction are of concern. As TTFields is a portable home-use device it involves no treatment-related travel thereby avoiding additional hospital exposure. Furthermore, the most common skin-related adverse events can be managed in the home via telemedicine. Clinical trial enrollment with TTFields have continued relatively unaffected. The expert panel further concluded that it is currently too early to see the full impact of COVID-19 on GBM patients and strongly recommended establishing support via the cancer and COVID-19 registries (**Table 1**).

**Table 1:** Summary of Key Points: Based on expert panel opinion.

Summary of Key Points
Safer practices for TTFields patient assessment and education as well as safer device delivery and replacement have been instituted to meet the physical distancing recommendations during the COVID-19 pandemic.
For established patientsTTFields can be safely continued during the COVID-19 pandemic
For new patientsTTFields can be safety initiated during the COVID-19 pandemic
TTFields clinical trials participation should be continued and encouraged provided there is appropriate clinical/research support during the COVID-19 pandemic.
Continuous assessment of treatment practices and outcomes for GBM patients during the COVID-19 pandemic is of critical importance to the field of Neuro-Oncology. Affiliation with the established COVID-19 and cancer registries is important to capture these data.

We recognize that there are non-safety issues that might limit brain tumor patients access to use of the TTFields device during the COVID-19 pandemic. One important limitation might be due to out-of-pocket expense to the patient and/or the already challenged healthcare system during this crisis. While the cost impact on therapeutic access was outside of the scope of this panel discussion, we agree this is an important issue to consider and should be studied further.

## Data Availability Statement

The original contributions presented in the study are included in the article material. Further inquiries can be directed to the corresponding author.

## Author Contributions

NG, JD, RH, AH, VP, DT, EW, and MG were involved with the panel discussion and the development of the expert opinion. NG, JB-S, JD, RH, AH, PH, CK, VP, DT, EW, and MG were involved with the analysis and interpretation of the expert opinion, and were involved in all stages of the development of this manuscript including its final approval. All authors contributed to the article and approved the submitted version.

## Funding

The May 2020 Advisory Board was funded by Novocure Inc.

## Conflict of Interest

PH was employed by Novocure Inc.

NG, JB-S, JD, RH, AH, PH, VP, DT, EW, and MG served on the May 2020 Advisory Board for Novocure, and declare that they received funding from Novocure for their attendance. CK received funding in support of its overall program in 2020 from Novocure. MG has received an honorarium from Novocure, is on a Novocure advisory board, and received funding from Novocure for a phase I/II trial. AH has received travel support for medical meeting to present trial results (paid to his institution).

The authors declare that this study received funding from Novocure Inc. The funder had the following involvement with the study: Novocure provided editorial support only for the development of this manuscript: which is a summary of the authors opinions, discussions, and recommendations that were garnered from the Advisory Board.
